# Evolution of Resistance in Cancer: A Cell Cycle Perspective

**DOI:** 10.3389/fonc.2019.00376

**Published:** 2019-05-09

**Authors:** Kağan Dökümcü, Ramin M. Farahani

**Affiliations:** ^1^Department of Life Sciences, Faculty of Medicine and Health, University of Sydney, Sydney, NSW, Australia; ^2^IDR/Westmead Institute for Medical Research, Westmead, NSW, Australia

**Keywords:** mutagenesis, cell cycle, quiescence, tolerance, resistance

## Abstract

Resistance of neoplastic cells to therapy is considered a key challenge in the treatment of cancer. Emergence of resistance is commonly attributed to the gradual mutational evolution of neoplastic cells. However, accumulating evidence suggests that exogenous stressors could significantly accelerate the emergence of resistant clones during the course of treatment. Herein, we review molecular mechanisms that regulate the evolution of resistance in a tumor with particular emphasis on the role of cell cycle.

## Introduction

Emergence of resistant cells in a neoplastic population complicates the course of treatment and leads to recurrence of cancer. This is because resistant clones can efficiently adapt to exogenous stressors including therapeutic agents. Despite being widely acknowledged as a central issue in the treatment of cancer, mechanisms that facilitate emergence of resistant neoplastic cells remain largely unknown. It is commonly believed that gradual evolution of a neoplastic genome by mutational changes drives the phenotypic heterogeneity in a uniform population of cells. This paradigm assumes a lack of directionality owing to the random nature of mutations. This notion, however, conflicts with evidence that suggests exogenous stressors could accelerate and direct emergence of resistant neoplastic cells. Keats et al. demonstrated that treatment of multiple myeloma triggers a clonal competition that in consequence leads to oscillatory dominance of resistant subclones (1). Herein, we review the molecular mechanisms that facilitate and direct emergence of resistance in a neoplastic population. We particularly focus upon the role of endogenous mechanisms of adaptation to stressors in instructing cancer resistance.

## Tolerance and Resistance: Separate but Intertwined

**Figure 1:**
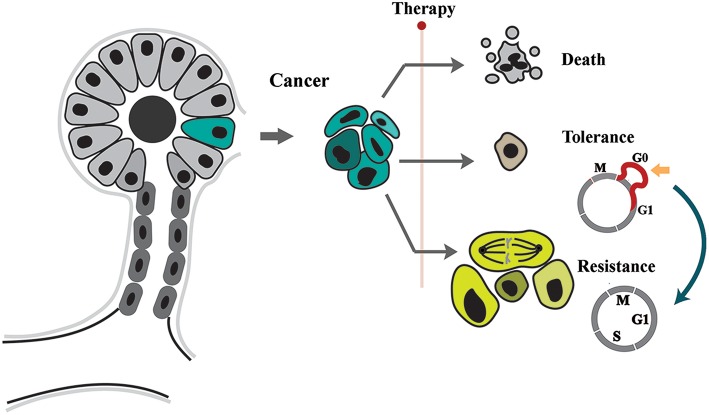
Tolerance and resistance drive emergence of neoplastic adaptation to stressors. The neoplastic cells respond to applied stressor by apoptosis, tolerance, or resistance. While in tolerance mode cells remain in quiescence by expansion of G0, resistant cell proliferate despite presence of stressor.

Adaptation of neoplastic cells to therapy can be induced by two parallel and yet distinct mechanisms (Figure 1). “Tolerance” to therapy occurs when neoplastic cells simply transition into a dormant or quiescent state due to applied therapies and survive the stressors but do not proliferate (2). This phenomenon is similar to bacterial tolerance induced by application of antibiotics (3). In contrast to tolerance, resistant cells continue to proliferate despite presence of cytotoxic or genotoxic stressors. Resistance requires altered interpretation of exogenous stressors by neoplastic cells. Such capacity is usually acquired by mutations that reduce the effective concentration of the stressors (4).

The two proposed modes of neoplastic adaptation can be distinguished phenotypically during the course of treatment. Tolerance leads to a phenomenon commonly known as tumor dormancy (2, 5). During dormancy, the “occult tumor” is clinically undetectable (5). In the subsequent phase, evolution of resistance leads to the relapse of cancer and concomitant unresponsiveness to applied therapies.Despite being separate entities, there is some evidence that tolerance can be a prelude to mutational evolution and resistance (6, 7). This proposal is not unprecedented. In a parallel scenario, it was demonstrated that emergence of antibiotic resistance is accelerated in a background of tolerance (i.e., quiescence) (8). This raises important questions about the molecular signature of the tolerance program and how it facilitates subsequent emergence of resistance.

## Reprogramming of Cell Cycle Facilitates Tolerance of Stressors

**Figure 2:**
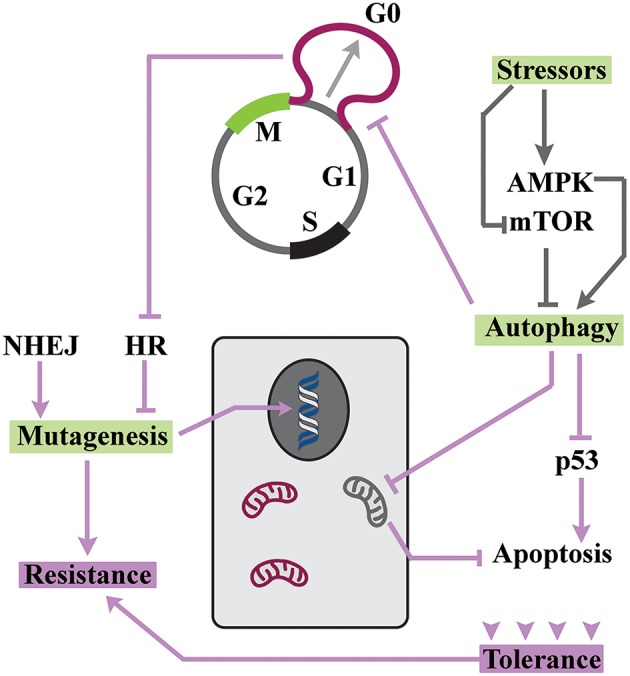
Resistance can develop in a background of tolerance. The enhanced autophagic flux drives adaptation to stressors and also arrests the cycle at G0. Due to inhibition of HR, DNA repair by NHEJ generates a hypermutability state that is tolerated owing to enhanced autophagic flux.

Tolerance is induced by a reversible exit from cell cycle during transition from mitosis to G1 phase (2). This phase, commonly known as G0 (9), is defined based on the sensitivity of cell cycle to nutritional stressors. During G0 and prior to the “restriction point” (10) nutritional information is processed by cycling cells and integrated into multiple signaling cascades that license progression into G1 phase under optimal availability of nutrients (11). Suboptimal nutritional condition, on the other hand, leads to transient arrest at G0 (11). Lengthening G0 requires global changes in metabolic activity of cycling cells (Figure 2). In the nutrient limiting condition, reduced ratio of ATP/AMP leads to activation of the AMPK (AMP-activated protein kinase) cascade (12) and inhibition of mammalian target of rapamycin (mTOR) signaling (13). In consequence, autophagy is activated (14) to balance the energetic demands of stressed cells via autocatalytic activity (15) and energy-consuming protein synthesis is inhibited (16). The enhanced autophagic flux leads to transient arrest of cell cycle (17) while repressing the induction of a senescence program (18). This occurs due to convergence of autophagy and proteolysis (19). Key regulators of autophagy, such as glycogen synthase kinase-3β (Gsk-3β), also drive degradation of cyclin D1 (20). Autophagic elimination of cyclin-D1 (21), along with other parallel events in pro-catalytic G0 phase, transiently abolishes progression of cell cycle.

Since autophagy improves the adaptive capacity of cells (22), it is not surprising that cell cycle arrest at pro-autophagic G0 leads to amplified tolerance of neoplastic cells (23). In fact, autophagy has been suggested to be the main gateway to tumor dormancy (24). The improved adaptability of cells in pro-autophagic G0 is mainly related to changes that are dictated by energetic demands. Signaling by AMPK leads to activation of ULK1 and ULK2 and autophagic elimination of mitochondria (25). This pro-survival change reduces the number of mitochondria that accommodate several key apoptotic inducers (26) and renders quiescent cells more resistant to apoptotic stimuli. Senescence is another route for elimination of neoplastic cells (27). Notably, autophagic flux also inhibits pathways that induce senescence (18). Autophagic degradation of p53 (28), in particular, is a major contributor to the repression of senescence during quiescence (29). Yet, inhibition of p53 has another profound consequence and that is reduced apoptotic tendency of quiescent cells (30). Another important aspect of autophagy is that the molecular machinery that propels autophagosome and endosome formation is shared (31). Therefore, enhanced autophagic flux would reduce the capacity of cells for concomitant endocytosis and impair the uptake of cytotoxic agents by neoplastic cells (32).

The pathways that instruct adaptation to stressors are conserved from *Caenorhabditis elegans* to human (22). In fact, the conservation extends beyond metazoan animals. In addition to the canonical pathways described, progression of cell cycle into G1 in yeasts requires further licensing by a complex molecular network that senses the availability of nitrogen and phosphate sources (33). The cascade in regulated by a cyclin-dependent protein kinase, Pho85 (34). Under suboptimal nutritional conditions, inhibition of Pho85 leads to transient cell cycle arrest at G0 (33). It was recently demonstrated that human breast carcinoma cells utilize the activity of this ancient cascade to enhance tolerance to genotoxic stressors (35). MiRNA4673 is central to this tolerance mechanism. The miRNA enhances autophagy by targeting cdk18, the human homolog of yeast metabolic sensor Pho85 (34, 36). Inhibition of cdk-18 simulates a faux nutrient-deprivation condition and activates autophagy leading to improved tolerance of breast carcinoma cells by transient arrest at G0 (35). The activity of miR4673 has another significant consequence. The miRNA alters kinetics of DNA repair and increases mutability of the neoplastic cells (35).

## Tolerance is Conducive to Hypermutability

Multiple mechanisms are involved in repair of DNA damage (37). The two major pathways for repair of DNA double-stranded breaks are homologous recombination (HR) and non-homologous end joining (NHEJ) (38). While repair based on HR has a higher fidelity, NHEJ is more efficient in repairing DNA damage (repair time ≈30 min for NHEJ vs. >7 h for HR). Notably, DNA repair mechanisms vary according to the phase of cycle (39). Homologous recombination (HR) is, in particular, repressed during G0/early G1 (40). The inhibition of HR alters the balance in favor of error-prone non-homologous end-joining (NHEJ) (41). The shift to NHEJ during quiescence (G0) by inhibition of HR improves tolerance to exogenous stressors (42, 43). This is in part due to accelerated kinetics of NHEJ compared to HR (38). However, repair by error-prone NHEJ amplifies the mutability of quiescent cells. Such hypermutability is not just a bystander effect. Under stressful conditions, hypermutability is actively encouraged to accelerate evolution of novel traits that improve the adaptability of cells to exogenous stressors (44). Double-stranded breaks are reported to drive stress-induced mutagenesis (45). In addition to damage-related mutations, transposable elements are involved in the emergence of novel phenotypes by hypermutability (46). The parallel role of pro-survival mechanisms that improve tolerance of neoplastic cells to DNA damage during G0 should not be neglected. Autophagic depletion of p53 (28), for example, reduces pro-apoptotic signaling in stressed cells (30). Remarkably, depletion of p53 triggers hypermutability by reducing the fidelity of double-stranded break repair mechanisms (47). The evidence suggests that induction of autophagy during G0, not only improves tolerance by the mechanisms described, but also accelerates emergence of resistance by amplifying the mutability of neoplastic cells. This scenario is aligned to the proposed evolution of resistance in the background of tolerance (8). The resistant cells, upon re-entry into cell cycle, would trigger the relapse of a dormant cancer (6, 48).

## Therapeutic Implications

From a treatment perspective, tolerance-mediated resistance provides an operational framework for developing therapeutic strategies (5, 49, 50). One such strategy is to maintain neoplastic cells in a state of dormancy (49). This approach is termed “sleeping strategy.” In another approach, termed “awakening strategy,” neoplastic cells are stimulated to re-enter the cell cycle in order to improve the efficiency of anti-proliferative drugs (51). In addition to canonical pathways of G0 arrest, non-canonical cdk18-dependent cascade (36) may be targeted to enhance progression of neoplastic cells into interphase (35). However, as mentioned previously, progression into cycle of arrested cells may lead to emergence of resistant clones. A safer approach could be induction of senescence in quiescent cells by repression of autophagy (18).

## Concluding Remarks

The evidence provided suggests that tolerance and resistance may be interpreted as linked traits in a spectrum. Such connectivity would have implications for diagnosis and therapy in cancer patients. Given the role of cell cycle in induction of quiescence and coupling of the latter to hypermutability, novel therapeutics that target cell cycle dynamics may improve the outcome of cancer treatment.

## Author Contributions

RF conception of the work and final version approval. KD and RF extensive literature search and manuscript drafting and critical revision of the work.

### Conflict of Interest Statement

The authors declare that the research was conducted in the absence of any commercial or financial relationships that could be construed as a potential conflict of interest.
